# Novel Calcium Phosphate Cement with Metformin-Loaded Chitosan for Odontogenic Differentiation of Human Dental Pulp Cells

**DOI:** 10.1155/2018/7173481

**Published:** 2018-11-27

**Authors:** Wei Qin, Jia-Yao Chen, Jia Guo, Tao Ma, Michael D. Weir, Dong Guo, Yan Shu, Zheng-Mei Lin, Abraham Schneider, Hockin H. K. Xu

**Affiliations:** ^1^Department of Operative Dentistry and Endodontics, Guanghua School of Stomatology, Sun Yat-sen University, Guangdong Provincial Key Laboratory of Stomatology, Guangzhou, China; ^2^Department of Advanced Oral Sciences and Therapeutics, University of Maryland School of Dentistry, Baltimore, USA; ^3^Department of Oncology and Diagnostic Sciences, University of Maryland School of Dentistry, Baltimore, USA; ^4^Department of Pharmaceutical Sciences, School of Pharmacy, University of Maryland, Baltimore, USA; ^5^Center for Stem Cell Biology & Regenerative Medicine, University of Maryland School of Medicine, Baltimore, USA; ^6^Department of Mechanical Engineering, University of Maryland Baltimore County, Baltimore County, USA

## Abstract

Metformin is an old and widely accepted first-line drug for treating type 2 diabetes. Our previous studies demonstrate that metformin can stimulate the osteo/odontogenic differentiation of human-induced pluripotent stem cell-derived mesenchymal stem cells and human dental pulp cells (DPCs). Due to the rapid dilution of metformin from the defect area, the aim of this study was to develop a drug delivery system with controlled release of metformin to promote cell viability and odontogenic differentiation of DPCs favoring dentin regeneration. Calcium phosphate cement (CPC) containing chitosan and metformin as a scaffold was synthesized. DPCs were seeded onto the scaffold, and the viability and proliferation were evaluated at several time points. For osteogenic differentiation analysis, alkaline phosphatase (ALP) activity was tested, cells were stained with Alizarin Red, and the expression of odontogenic markers was evaluated by real-time polymerase chain reaction. DPCs remained viable and attached well to the CPC-chitosan composite scaffold. Moreover, the addition of metformin to the CPC-chitosan composite did not adversely affect cell proliferation, compared to that of CPC control. Our data further revealed that the novel CPC-chitosan-metformin composite enhanced the odontogenic differentiation of DPCs, as evidenced by higher ALP activity, elevated expression of odontoblastic markers, and strong mineral deposition. These results suggest that the new CPC-chitosan-metformin composite is a highly promising scaffold with the potential for tissue engineering applications including dentin regeneration.

## 1. Introduction

Dental pulp is often damaged by cariogenic infection, mechanical trauma, and clinical operative procedures. A conventional endodontic treatment for infected pulp tissues is root canal therapy, which involves the extirpation of the inflammatory pulp, but will decrease the fracture toughness and infection-resistance of the residual tooth because of malnutrition [1]. Dental pulp regeneration is one of the most promising therapeutic strategies, which would promote the repair of the pulp-dentin complex and improve the patient's life quality [2]. Notably, as biotechnology has progressed, there have been several attempts to establish new methods to better control the parameters of regenerative endodontic treatment procedures using tissue engineering strategies [3].

Tissue engineering is fundamentally based on the interaction among progenitor cells, biochemical molecules, and three-dimensional scaffold materials [4]. Human dental pulp cells (DPCs) as progenitor cells are an excellent cell source for dentin regeneration. DPCs are easy to harvest from donors including children losing their primary teeth and teenagers having their wisdom teeth removed, which are otherwise discarded as medical waste [5, 6]. In addition, DPCs are capable of odontogenic differentiation to form the dentin-pulp complex in dental pulp tissues [7, 8]. Biochemical factors are critical signalling molecules that instruct the DPCs to achieve pulp regeneration. Our previous studies demonstrated that the small molecule compound metformin has osteo/odontogenic effects by promoting the differentiation of human-induced pluripotent stem cell- (hiPSC-) derived mesenchymal stem cells (MSCs) and DPCs [9, 10]. Although metformin is important for the differentiation of DPCs with its ability to enhance odontogenic differentiation, the application of metformin was limited in dentin regeneration because of its rapid dilution from the defect area leading to inefficient tissue formation [11]. Therefore, it is important to achieve sustained local release of metformin to the dental pulp exposure site.

Several studies have incorporated growth factors into calcium phosphate cement (CPC) [12–14]. However, the strength of the protein-releasing CPC was significantly lower than that without proteins [15]. Our previous studies have shown that a reinforced CPC composite containing chitosan is an effective carrier and delivery vehicle for proteins [13] because chitosan can provide good mechanical strength and toughness to the scaffold [16]. However, to date, there has been no report of developing a CPC-chitosan-metformin composite. Therefore, the aim of this study was to develop a novel CPC-chitosan-metformin composite and investigate its effect on cell viability, proliferation, the expression of odontogenic genes, and mineral matrix deposition. The results of our study will provide a foundation for the future use of CPC-chitosan-metformin composite for cell-based dentin and other tissue regeneration therapies.

## 2. Materials and Methods

### 2.1. Fabrication of CPC-Chitosan-Metformin Scaffold

CPC powder consisted of tetracalcium phosphate [TTCP: Ca_4_(PO_4_)_2_O] and dicalcium phosphate anhydrous (DCPA: CaHPO_4_). Briefly, TTCP powder was formed via the solid-state reaction of DCPA and CaCO_3_ (both from J. T. Baker, Phillipsburg, NJ), which were mixed and heated in a furnace (Lindberg, Watertown, WI) at 1500°C for 6 hours. The heated mixture was quenched to room temperature in a desiccator and then ground in a ball mill (Retsch PM4, Brinkman, NY) to obtain particles with a 5 *μ*m median particle size. DCPA was ground in ball mill with 95% ethanol to obtain a powder with a median particle size of 1 *μ*m. TTCP and DCPA were mixed at 1 : 3 molar ratio to form the CPC powder. The CPC liquid contained water and chitosan. Chitosan is a natural polymer and is often used as scaffold in bone tissue engineering because of its biocompatibility, low toxicity, and degradability by enzymes [17]. First, 10, 30, and 50 *μ*g of metformin were, respectively, dissolved in water. Second, chitosan lactate was mixed with water containing various doses of metformin and dissolved in water at chitosan/(chitosan + metformin + water) mass fraction of 15% to form the CPC liquid. The 15% mass fraction was used because it yielded a good strength for CPC in a previous study [13]. Third, CPC paste was formed by mixing the sterile CPC powder with the CPC liquid at a CPC powder to liquid ratio of 3 to 1 by mass. The paste was placed in a mold with a diameter of 10 mm and a thickness of 1 mm and incubated in a humidor for 24 hours at 37°C. Five groups of specimens were thus fabricated:

1. CPC + 0% chitosan (CPC control)
2. CPC + the 15% chitosan liquid without metformin (CPC + CN control)
3. CPC + the 15% chitosan liquid + 10 *μ*g metformin in each specimen (CPC + CN + 10Met)
4. CPC + the 15% chitosan liquid + 30 *μ*g metformin in each specimen (CPC + CN + 30Met)
5. CPC + the 15% chitosan liquid + 50 *μ*g metformin in each specimen (CPC + CN + 50Met)

### 2.2. Metformin Release from CPC Scaffold

Carbon 14 [^14^C]-labeled compounds are often used to determine drug release amounts. The release of metformin was assessed following our previous method [18, 19]. Briefly, 10, 30, and 50 *μ*g of metformin (containing 1/10 [^14^C]-metformin) were dissolved in 15% chitosan to form the metformin + chitosan liquid. The powder and liquid portions were mixed under sterile conditions by hand spatulation. [^14^C] metformin-loaded scaffold was placed in 24-well plates containing 1 mL of PBS in an incubator at 37°C. At each time period, the microsphere suspension was centrifuged, and the PBS without microspheres was collected for [^14^C]-metformin concentration analysis. 100 *μ*L PBS was transferred to the scintillation tube containing 3 mL Biodegradable Counting Cocktail buffer (Fisher Scientific Inc., Pittsburgh, PA). Radioactivity was counted by a multipurpose scintillation counter (Beckman LS6500 Counter, Brea, CA).

### 2.3. Cell Culture

DPCs were isolated and characterized as described previously [20]. Dental pulp tissues were obtained from explants of clinically healthy dental pulps from human adult third molars that were removed from individuals undergoing tooth extraction for orthodontic treatment. The procedure was approved by the Institutional Review Board of the University of Maryland Baltimore. Briefly, pulp tissues were minced and digested in a solution of 3 mg/mL of collagenase type I and 4 mg/mL dispase for 30–60 min at 37°C. Cell suspension was obtained by passing the digested tissue through a 70 *μ*m cell strainer. The cells were pelleted and seeded in culture dishes and incubated in *α*-MEM supplemented with 20% FBS, 100 units/mL penicillin G, 100 mg/mL streptomycin, and 50 mg/mL ascorbic acid (Sigma-Aldrich, St. Louis, MO) at 37°C in 5% CO_2_. Nonadherent cells were removed 48 h after the initial plating. The medium was replaced every 3 days. When primary culture became subconfluent after approximately 1–2 weeks, cells were collected by trypsinization and subcultured at 5000 cells per cm^2^ in growth medium.

### 2.4. Flow Cytometry Analysis

To analyze the cell surface antigen expressions, the cells from the second passage were harvested by trypsin/EDTA treatment for 4 min at 37°C then washed twice with PBS. 1 × 10^5^ cells/tube were incubated with the conjugated antibody for 20 min on ice in the dark. The cell suspensions were washed twice, resuspended in 2% FBS/PBS, and analyzed using a flow cytometry cell sorting Vantage cell sorter (BD Biosciences, San Jose, CA). Data were analyzed using the FACS software (FlowJo LLC, Ashland, OR). The following conjugated antibodies were used: STRO-1-PE (Santa Cruz Biotech, sc-47733, Santa Cruz, CA), CD-29-PE (BD Biosciences, Cat. No. 557332, San Jose, CA), CD-90-PE (BD Biosciences, Cat. No. 555596), CD105-FITC (BD Biosciences, Cat. No. 561443), CD-34-PE (BD Biosciences, Cat. No. 560941), CD-45-PE (Santa Cruz Biotech, sc-28369), FITC-conjugated IgG control antibody (BD Biosciences, Cat. No. 555748), and PE-conjugated IgG control antibody (BD Biosciences, Cat. No. 555749). Isotype control antibodies were used as negative controls.

### 2.5. Cell Viability Assays

DPCs were seeded on a disk with a diameter of 10 mm and a thickness of 1 mm in 24-well plates at a cell seeding density of 1.5 × 10^5^ cells/well. At 1 and 7 days, cells were stained by live/dead viability assay kit (Invitrogen, Carlsbad, CA) as previously described [21]. Cells were washed with PBS, followed by incubation with the dye. Live cells were stained green with 2 mM calcein AM and dead cells were marked red with 4 mM ethidium homodimer-1 (EthD-1), and they were examined using epifluorescence microscopy (Eclipse TE2000-S, Nikon, Melville, NY). The percentage of live cells and the live cell density were calculated as previously described [21].

### 2.6. Cell Proliferation Assays

Cell proliferation was measured by the Cell Counting Kit-8 assay (CCK-8, Dojindo, Tokyo, Japan). DPCs were seeded on disks with a diameter of 10 mm and a thickness of 1 mm in 24-well plates at a density of 1.5 × 10^5^ cells/well, and cells were incubated for 1, 3, 5, and 7 d at 37°C and 5% CO_2_. The culture medium was replaced with fresh medium every 2 days. At each time point, a total of 450 *μ*L of DMEM and 50 *μ*L of CCK-8 solution were added to each well and incubated for 2 h. After this incubation, 100 *μ*L of the supernatant was transferred into a 96-well plate and read at 450 nm using a SpectraMax M5 plate reader (Molecular Devices, Sunnyvale, CA) according to the manufacturer's protocol. At least three independent experiments were performed, each of which was performed in triplicate.

### 2.7. ALP Assay

Cells were seeded at a density of 1.5 × 10^5^ cells/well with CPC scaffold in 24-well plates. ALP activity was measured at 1, 7, and 14 d using an ALP kit (Wako, Tokyo, Japan) following the manufacturer's instructions. Samples were washed twice in PBS before adding 0.5 mL cell lysis buffer containing 0.2% Triton X-100 (Sigma-Aldrich) with 10 mM Tris (pH 7.0) and 1 mM EDTA (Sigma-Aldrich) onto the CPC disks. They were incubated for 20 minutes and transferred to −80°C freezer for 30 minutes and thawed at room temperature for 30 minutes. The freeze-thawing procedure was performed twice to lyse cells and collect all the ALP samples. Finally, the test sample was transferred to a 96-well plate, and its activity was measured at 520 nm concurrently with standard samples. The ALP activity was calculated against the total cellular protein concentrations to obtain [pNpp (*μ*M/min)]/[Protein (mg)]. The amount of protein in each sample was measured by a BCA protein assay kit (Thermo Scientific, Waltham, MA). For the ALP activity assays, each sample was performed in triplicate and the results were repeated in at least three independent tests.

### 2.8. Reverse Transcriptase PCR (RT-PCR) and Real-Time Quantitative PCR (qPCR)

The expression levels of dentin sialophosphoprotein (DSPP), dentin matrix protein-1 (DMP-1), runt-related transcription factor-2 (Runx2), and osteocalcin (OCN) mRNA were determined by SYBR green real-time reverse transcription-PCR (RT-PCR) as previously described [22–24]. Total RNA was extracted using Trizol reagent. Quantitative determination of RNA levels was performed in triplicate in three independent experiments. Real-time PCR and data collection were performed with an ABI PRISM 7500 sequence detection system. The housekeeping gene GAPDH was used as an internal control to normalize the expression levels of different genes. For each primer set, the melting curves were performed to ensure that a single peak was produced. The data for gene expression were analyzed using the △△Ct method. The primers used for the amplification of the indicated genes are listed in Table 1.

### 2.9. Alizarin Red Staining (ARS) of Mineral Synthesis by DPCs

ARS was performed to evaluate the mineralization by DPCs [25]. At days 1 and 21, disks were fixed using 10% formaldehyde for 30 minutes, washed by PBS, and stained with 2% Alizarin Red S (Millipore, Billerica, MA) for 45 minutes. To quantify the mineral deposition, the stained minerals were dissolved by 10% cetylpyridinium chloridemonohydrate, then the extracted stain was transferred to a 96-well plate. The absorbance at 562 nm was measured using a microplate reader, as previously described [26, 27].

### 2.10. Statistical Analyses

Statistical analysis was performed with one-way analysis of variance (ANOVA), followed by post hoc LSD (least significant difference) tests using the Statistical Package for the Social Sciences (SPSS 20.0). All data were expressed as mean ± standard deviation (SD). Kolmogorov–Smirnov test and Levene's test were first performed to confirm the normality and equal variance of the data. A *P* < 0.05 was considered statistically significant.

## 3. Results

### 3.1. Identification of Stem Cell Phenotypic Markers in Primary DPCs

**Figure 1:**
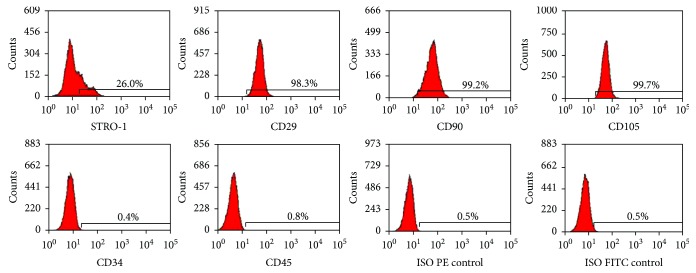
DPC phenotype by flow cytometry. The expression of a series of cell surface markers associated with the MSC phenotype was investigated using flow cytometry. Analysis of molecular surface antigen markers in DPCs by flow cytometry indicated that the cells were negative for CD34 and CD45, whereas they were positive for STRO-1, CD29, CD90, and CD105.

STRO-1^+^, CD29^+^, CD90^+^, and CD105^+^ have been shown to exhibit mesenchymal stem cell (MSC) properties, and these markers have been used to identify DPCs [28]. The surface markers of DPCs were analyzed by flow cytometry. Consistent with other MSCs, the majority of DPCs were negative for CD34 and CD45. The culture population contained 26.0% STRO-1-positive cells, 98.3% CD29-positive cells, 99.2% CD90-positive cells, and 99.7% CD105-positive cells (Figure 1). These results indicate that the DPCs were mesenchymal progenitors.

### 3.2. Metformin Release from Metformin-Loaded CPC Scaffold

**Figure 2:**
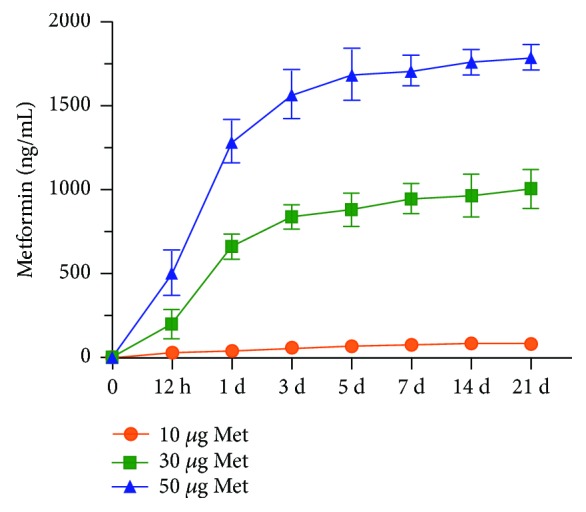
Metformin release from CPC-chitosan scaffold. CPC + CN + 50Met composite exhibited greater release in the first 12 h as compared to CPC + CN + 10Met and CPC + CN + 30Met. Metformin from CPC-chitosan scaffolds had sustained release over 21 days. CPC: calcium phosphate cement.

The drug release of metformin-CPC scaffold was analyzed using [^14^C] label. The release profile showed a typical fast release initially, reaching an accumulative release of about 29.9, 199.4, and 505.1 ng/mL in the first 12 h for samples containing 10, 30, and 50 *μ*g of metformin, respectively. Then, the CPC continued to release metformin at a sustained rate during the next 21 days (Figure 2). The release amount was related to the initial loading concentrations of metformin. Based on the initial burst release of metformin and followed by continuous release, the 50 *μ*g metformin was selected in all further experiments. These results showed that metformin release from CPC-chitosan scaffold was sustained and relatively long lasting, to meet the requirement of a drug carrier to last for 2–3 weeks in order to stimulate the odontogenic differentiation of DPCs.

### 3.3. Viability and Cell Proliferation of DPCs on CPC Scaffold

**Figure 3:**
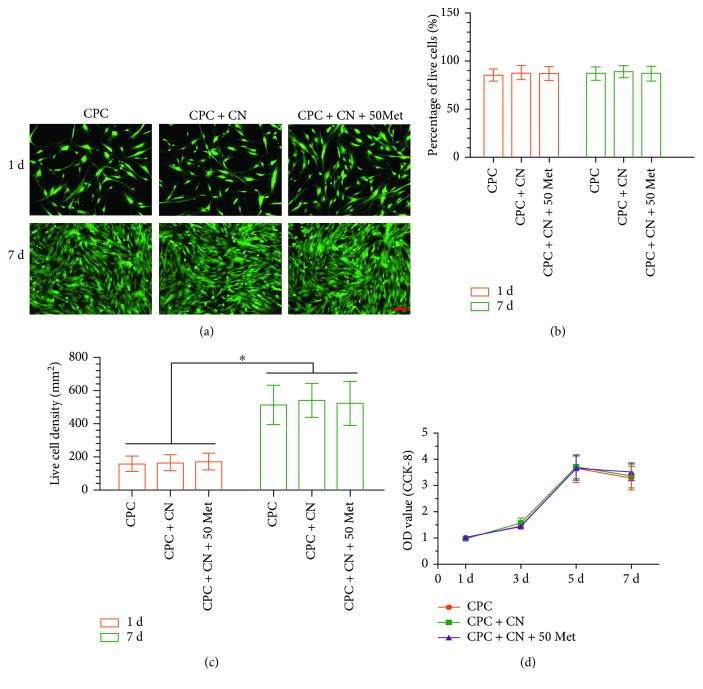
Viability and proliferation of DPCs attached on CPC scaffold surface. (a) Representative live/dead images of metformin-treated DPCs at days 1 and 7, with live cells stained green and dead cells shown in red. In all three groups, live cells were abundant, and dead cells were few (scale bar = 50 *μ*m). (b) The percentage of live cells of DPCs was around 89%. (c) All groups exhibited an increasing live cell density; ^∗^*P* < 0.05. (d) CPC + CN + 50Met had no effect on cell proliferation. Data represent mean ± SD of three independent experiments with triplicates.

Representative live/dead staining images are shown in Figure 3(a). There were numerous live cells (stained green) and a few dead cells (stained red). The cell number increased from day 1 to day 7 due to proliferation. In Figure 3(b), the percentages of live cells on CPC control, CPC + CN control, and CPC + CN + 50Met groups were greater than 89% and were not significantly different from each other (*P* > 0.05). As shown in Figure 3(c), the live cell density increased with time due to proliferation, with no significant difference among the three groups (*P* > 0.05). The OD value suggests that there were no significant differences in the cell proliferation for the three groups (*P* > 0.05) (Figure 3(d)). Overall, no noticeable difference was observed among the three groups, indicating that the metformin in the CPC scaffold was not cytotoxic to DPCs.

### 3.4. Alkaline Phosphatase (ALP) Activity

**Figure 4:**
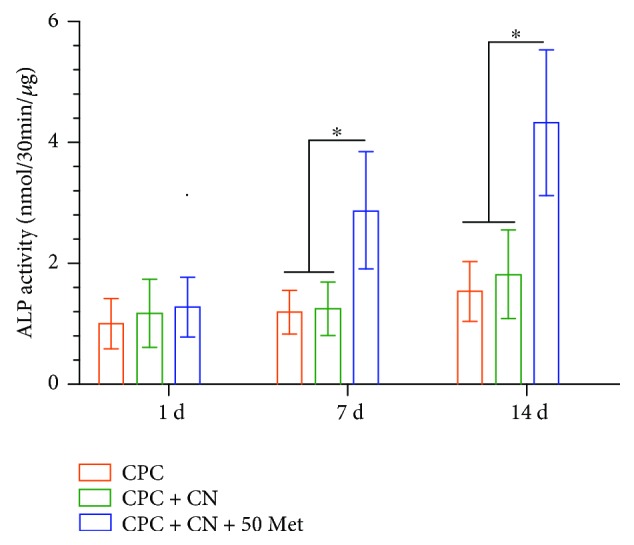
Alkaline phosphatase (ALP) assay. ALP activity was measured at days 1, 7, and 14. A significant increase in ALP activity was observed in the CPC + CN + 50Met group compared with the CPC control and CPC + CN control (^∗^*P* < 0.05). Data represent mean ± SD of three independent experiments with triplicates.

The ALP activity of DPCs is plotted in Figure 4. The addition of metformin to CPC resulted in a significant increase in ALP activity compared to CPC control and CPC + CN control. The ALP activity of CPC containing no chitosan was similar to that of CPC + CN control (*P* > 0.05), indicating that the addition of chitosan to CPC had no effect on ALP activity. These results demonstrate the great potential of DPCs + CPC + metformin construct as an ideal “stem cell-scaffold-small molecule” system for dentin regeneration.

### 3.5. Odontogenic Differentiation

**Figure 5:**
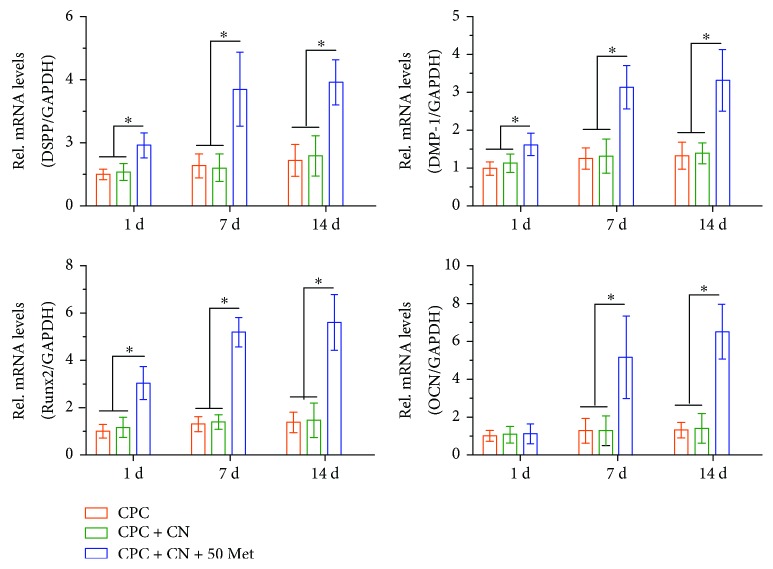
DPC odontogenic differentiation on CPC control, CPC + CN control, and CPC + CN + 50Met. The mRNA expression levels of DSPP, DMP-1, Runx2, and OCN were determined using qRT-PCR. The mRNA expression levels of DSPP, DMP-1, Runx2, and OCN significantly increased in the CPC + CN + 50Met compared with the CPC control and CPC + CN control (^∗^*P* < 0.05). GAPDH was used as an internal control. Data represent mean ± SD of three independent experiments with triplicates.

To further investigate the effects of CPC containing metformin on odontogenic differentiation of DPCs, the DPC odontogenic differentiation in the three groups was evaluated. Metformin significantly increased the mRNA expression of odontoblastic gene markers, including DSPP, DMP-1, Runx2, and OCN mRNA (Figure 5).

### 3.6. Mineralization by DPCs

**Figure 6:**
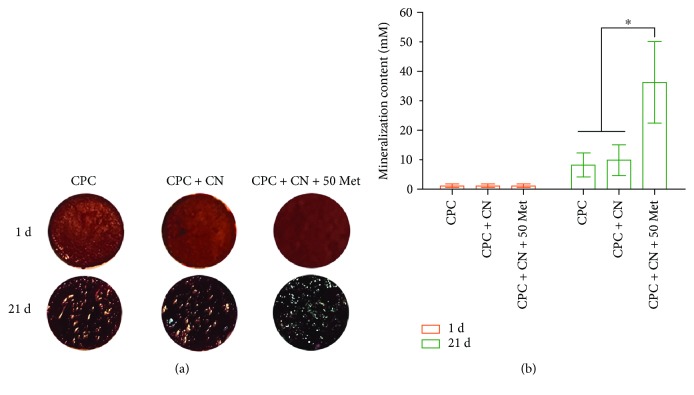
Mineralization by DPCs analyzed by Alizarin Red S staining. (a) After the cells were cultured for 21 days in osteogenic differentiation media, the mineral matrix exhibited a denser and darker red staining with increasing culture time. (b) Quantification of Alizarin Red S staining. The graph shows that the amount of calcium was significantly greater in the CPC + CN + 50Met than in the CPC control and CPC + CN control (^∗^*P* < 0.05). Data represent mean ± SD of three independent experiments with triplicates.

Next, we investigated the formation of mineralized nodules, an index of terminal odontoblastic differentiation, in DPCs after 21 days of incubation with metformin. The mineralized extracellular deposits produced by DPCs on scaffold surfaces are shown in Figure 6(a). The staining of the synthesized bone mineral matrix became denser and darker, reflecting that the mineral synthesis was increased significantly from day 1 to 21. Data from the cetylpyridinium chloride monohydrate are plotted in Figure 6(b). For each group, the mineral amount synthesized by the cells increased dramatically from day 1 to 21 (*P* < 0.05). These results indicate that CPC + CN + 50Met had the greatest bone mineral synthesis by the cells than other groups (*P* < 0.05).

## 4. Discussion

DPCs were selected as suitable MSCs for dentin regeneration, mainly for the treatment of pulp exposures [29]. Although bone marrow MSCs have become the main cell source for bone tissue engineering [30], bone marrow donation is an invasive surgical procedure. Previous studies demonstrated the high capacity of DPCs for odontogenic differentiation and potential to form dentin-pulp complexes when transplanted into immunocompromised mice [31]. These cells exhibited high proliferation rate, great differentiation potential, and expression of mesenchymal and embryonic stem cell markers. In the present study, DPCs strongly expressed MSC markers, indicating that DPCs maintained the stem cell characteristics and may have a high capacity for differentiation.

Currently, the application of tissue engineering concepts for the development of biodegradable scaffolds capable of driving dental pulp cell migration and differentiation has been the focus of dentin regeneration [32]. Loading scaffolds with small molecule compounds to facilitate tissue regeneration has already been studied and reported in the tissue engineering literature. CPC has been increasingly used in dental, craniofacial, and orthopaedic repairs as bone graft substitutes [33]. However, its use is limited by the relatively poor strength properties. It has been reported that chitosan-based scaffolds showed no cytotoxicity toward various cell types [34]. Chitosan incorporation into CPC could improve the load-bearing capability of the scaffold. For example, in a previous study, the CPC-chitosan scaffold had a flexural strength of (19.5 ± 1.4) MPa, higher than the (8.0 ± 1.4) MPa of CPC control without chitosan [35]. In addition, several studies indicated that metformin has osteogenic effects by promoting the differentiation of MSCs and preosteoblasts [36, 37]. Therefore, in our current study, metformin was added to the chitosan solution, which was then mixed with CPC powder to form the CPC paste. The release profile from CPC in 10, 30, and 50 *μ*g concentration of metformin per specimen displayed a sustained manner up to 21 days. Nevertheless, there was a significantly smaller amount of metformin released from 10 *μ*g and 30 *μ*g specimens, compared to the 50 *μ*g specimens. The sustained release of metformin from these specimens was likely due to the distribution of metformin into chitosan, whereas the initial burst release was likely due to the binding of metformin to the CPC surface. Furthermore, the bioactivity of the released metformin is very important; our results showed that the cell viability at 1 day and 7 days was similar for all CPC-based materials. Live cell density for all CPC-based materials was also similar. Therefore, CPC + CN and CPC + CN + 50Met did not adversely affect the cell proliferation when compared with CPC control, which was approved by the FDA for craniofacial repairs. Hence, by comparing with the results of previous studies [16], our current study showed an acceptable biocompatibility for CPC-metformin.

After confirmation of the effects of metformin on DPC viability and cell proliferation, we examined the odontogenic differentiation ability of cells seeded on CPC-chitosan scaffolds with and without metformin. ALP activity is most often used as an early marker of odontoblastic differentiation [38] and plays an important role in cell mineralization. In the current study, the CPC + CN + 50Met construct yielded a significantly higher ALP activity than the CPC control and CPC + CN control. Consistent with this finding, more mineralized nodules were also observed in DPCs cultured with CPC + CN + 50Met group at the late stage of odontogenic differentiation.

Moreover, the gene expression levels of the related odontogenic gene markers such as DSPP, DMP-1, Runx2, and OCN were measured to elucidate the effects of the CPC + CN + 50Met composite on the odontogenic differentiation in vitro. DSPP was originally thought to be a dentin-specific marker. Although several studies have also indicated its expression in bone [39, 40], DSPP remains to be an important marker for odontogenic differentiation. In addition, DMP-1 is a key regulator of odontogenic differentiation and the formation of mineralization and the dentin tubular system [41]. Furthermore, Runx2, of the runt domain gene family, is a key transcription factor that controls bone and tooth formation by regulating the osteo/odontogenic differentiation [42, 43]. Moreover, OCN can be released by odontoblasts and is present in the dentin matrix, and it is also used as a reparative molecule within the dental pulp [44–46]. In the present study, DSPP, DMP-1, Runx2, and OCN mRNA levels were all upregulated in DPCs cultured with the CPC + CN + 50Met group. However, no effect was observed by metformin on the mRNA level of OCN during the early stages of treatment. These results may be attributed to the fact that OCN was expressed at the later stage of cell mineralization [47]. These results suggest that metformin can be considered to be a potential biochemical factor for odontogenic differentiation of DPCs when cultured in CPC-chitosan scaffold.

However, the present work possesses several limitations. The accurate and quantitative control of metformin release from scaffolds is difficult. There are controversies surrounding even well-investigated small molecules, and studies based on different models often yield different results [48, 49]. Thus, a further in-depth investigation should be performed to identify the mechanism of metformin on odontogenesis stimulation at the molecular level. In addition, further in vivo experiments should also be performed using an animal model to demonstrate dentin and other tissue regeneration for future clinical applications.

## 5. Conclusions

The present study showed for the first time that the incorporation of metformin into CPC-chitosan composite significantly enhanced odontogenic differentiation of DPCs, without negatively altering the cell viability and proliferation. CPC-chitosan composite scaffold is promising to be a moderate load-bearing matrix for cell delivery [35] and dentin regeneration, with the potential to serve as a delivery vehicle for metformin to promote the regeneration of dentin as well as other types of tissues. These findings will provide critical insights towards the future use of DPCs/chitosan/metformin combinations in dentin pulp and other tissue engineering applications.

## Figures and Tables

**Table 1:** The sequences of specific primers for real-time PCR operation.

Gene	Forward	Reverse
DSPP	GCCACTTTCAGTCTTCAAAGAGA	GCCCAAATGCAAAAATATGTAA
DMP-1	AAAATTCTTTGTGAACTACGGAGG	GAGCACAGGATAATCCCCAA
Runx2	GACTGTGGTTACCGTCATGGC	ACTTGGTTTTTCATAACAGCGGA
OCN	CTCACACTCCTCGCCCTATT	TTGGACACAAAGGCTGCAC
GAPDH	TCAACGACCCCTTCATTGAC	ATGCAGGGATGATGTTCTGG

## Data Availability

The data used to support the findings of this study are available from the corresponding author upon request.

## References

[1] Sjogren U., Hagglund B., Sundqvist G., Wing K. (1990). Factors affecting the long-term results of endodontic treatment. *Journal of Endodontics*.

[2] Simon S. R. J., Berdal A., Cooper P. R., Lumley P. J., Tomson P. L., Smith A. J. (2011). Dentin-pulp complex regeneration: from lab to clinic. *Advances in Dental Research*.

[3] Gong T., Heng B. C., Lo E. C. M., Zhang C. (2016). Current advance and future prospects of tissue engineering approach to dentin/pulp regenerative therapy. *Stem Cells International*.

[4] Langer R., Vacanti J. (1993). Tissue engineering. *Science*.

[5] Atari M., Gil-Recio C., Fabregat M. (2012). Dental pulp of the third molar: a new source of pluripotent-like stem cells. *Journal of Cell Science*.

[6] Huang A. H.-C., Chen Y.-K., Chan A. W.-S., Shieh T.-Y., Lin L.-M. (2009). Isolation and characterization of human dental pulp stem/stromal cells from nonextracted crown-fractured teeth requiring root canal therapy. *Journal of Endodontics*.

[7] Kuang R., Zhang Z., Jin X. (2016). Nanofibrous spongy microspheres for the delivery of hypoxia-primed human dental pulp stem cells to regenerate vascularized dental pulp. *Acta Biomaterialia*.

[8] Rosa V., Dubey N., Islam I., Min K. S., Nör J. E. (2016). Pluripotency of stem cells from human exfoliated deciduous teeth for tissue engineering. *Stem Cells International*.

[9] Wang P., Ma T., Guo D. (2018). Metformin induces osteoblastic differentiation of human induced pluripotent stem cell-derived mesenchymal stem cells. *Journal of Tissue Engineering and Regenerative Medicine*.

[10] Qin W., Gao X., Ma T. (2018). Metformin enhances the differentiation of dental pulp cells into odontoblasts by activating AMPK signaling. *Journal of Endodontics*.

[11] Kajbaf F., Bennis Y., Hurtel-Lemaire A. S., Andrejak M., Lalau J. D. (2016). Unexpectedly long half-life of metformin elimination in cases of metformin accumulation. *Diabetic Medicine*.

[12] Ruhé P. Q., Kroese-Deutman H. C., Wolke J. G. C., Spauwen P. H. M., Jansen J. A. (2004). Bone inductive properties of rhBMP-2 loaded porous calcium phosphate cement implants in cranial defects in rabbits. *Biomaterials*.

[13] Weir M. D., Xu H. H. (2010). Osteoblastic induction on calcium phosphate cement-chitosan constructs for bone tissue engineering. *Journal of Biomedical Materials Research Part A*.

[14] Blom E. J., Klein-Nulend J., Wolke J. G. C., Kurashina K., van Waas M. A. J., Burger E. H. (2002). Transforming growth factor-*β*1 incorporation in an *α*-tricalcium phosphate/dicalcium phosphate dihydrate/tetracalcium phosphate monoxide cement: release characteristics and physicochemical properties. *Biomaterials*.

[15] Ruhe Q. P., Hedberg E. L., Padron N. T., Spauwen P. H. M., Jansen J. A., Mikos A. G. (2003). rhBMP-2 release from injectable poly(DL-lactic-co-glycolic acid)/calcium-phosphate cement composites. *The Journal of Bone and Joint Surgery-American Volume*.

[16] Xu H. H. K., Simon C. G. (2005). Fast setting calcium phosphate–chitosan scaffold: mechanical properties and biocompatibility. *Biomaterials*.

[17] Costa-Pinto A. R., Reis R. L., Neves N. M. (2011). Scaffolds based bone tissue engineering: the role of chitosan. *Tissue Engineering Part B: Reviews*.

[18] Li Q., Guo D., Dong Z. (2013). Ondansetron can enhance cisplatin-induced nephrotoxicity via inhibition of multiple toxin and extrusion proteins (MATEs). *Toxicology and Applied Pharmacology*.

[19] al Jofi F. E., Ma T., Guo D. (2018). Functional organic cation transporters mediate osteogenic response to metformin in human umbilical cord mesenchymal stromal cells. *Cytotherapy*.

[20] Qin W., Huang Q.-T., Weir M. D. (2017). Alcohol inhibits odontogenic differentiation of human dental pulp cells by activating mTOR signaling. *Stem Cells International*.

[21] Zhao L., Weir M. D., Xu H. H. K. (2010). An injectable calcium phosphate-alginate hydrogel-umbilical cord mesenchymal stem cell paste for bone tissue engineering. *Biomaterials*.

[22] Wang P., Liu X., Zhao L. (2015). Bone tissue engineering via human induced pluripotent, umbilical cord and bone marrow mesenchymal stem cells in rat cranium. *Acta Biomaterialia*.

[23] Guo J., Qin W., Xing Q. (2017). TRIM33 is essential for osteoblast proliferation and differentiation via BMP pathway. *Journal of Cellular Physiology*.

[24] Yang X., Li Y., Liu X., Zhang R., Feng Q. (2018). In vitro uptake of hydroxyapatite nanoparticles and their effect on osteogenic differentiation of human mesenchymal stem cells. *Stem Cells International*.

[25] Liu X., Chen W., Zhang C. (2017). Co-seeding human endothelial cells with human-induced pluripotent stem cell-derived mesenchymal stem cells on calcium phosphate scaffold enhances osteogenesis and vascularization in rats. *Tissue Engineering Part A*.

[26] Chen W., Zhou H., Weir M. D., Bao C., Xu H. H. K. (2012). Umbilical cord stem cells released from alginate–fibrin microbeads inside macroporous and biofunctionalized calcium phosphate cement for bone regeneration. *Acta Biomaterialia*.

[27] Poh P. S. P., Seeliger C., Unger M., Falldorf K., Balmayor E. R., van Griensven M. (2018). Osteogenic effect and cell signaling activation of extremely low-frequency pulsed electromagnetic fields in adipose-derived mesenchymal stromal cells. *Stem Cells International*.

[28] Lv F.-J., Tuan R. S., Cheung K. M. C., Leung V. Y. L. (2014). Concise review: the surface markers and identity of human mesenchymal stem cells. *Stem Cells*.

[29] Yang I. S., Lee D. S., Park J. T., Kim H. J., Son H. H., Park J. C. (2010). Tertiary dentin formation after direct pulp capping with odontogenic ameloblast-associated protein in rat teeth. *Journal of Endodontics*.

[30] Phinney D. G., Prockop D. J. (2007). Concise review: mesenchymal stem/multipotent stromal cells: the state of transdifferentiation and modes of tissue repair-current views. *Stem Cells*.

[31] Gronthos S., Mankani M., Brahim J., Robey P. G., Shi S. (2000). Postnatal human dental pulp stem cells (DPSCs) in vitro and in vivo. *Proceedings of the National Academy of Sciences of the United States of America*.

[32] Piva E., Silva A. F., Nör J. E. (2014). Functionalized scaffolds to control dental pulp stem cell fate. *Journal of Endodontics*.

[33] Wang P., Zhao L., Chen W., Liu X., Weir M. D., Xu H. H. K. (2014). Stem cells and calcium phosphate cement scaffolds for bone regeneration. *Journal of Dental Research*.

[34] Chen W., Zhou H., Weir M. D., Tang M., Bao C., Xu H. H. K. (2013). Human embryonic stem cell-derived mesenchymal stem cell seeding on calcium phosphate cement-chitosan-RGD scaffold for bone repair. *Tissue Engineering Part A*.

[35] Weir M. D., Xu H. H. K. (2010). Culture human mesenchymal stem cells with calcium phosphate cement scaffolds for bone repair. *Journal of Biomedical Materials Research Part B: Applied Biomaterials*.

[36] Jang W. G., Kim E. J., Bae I. H. (2011). Metformin induces osteoblast differentiation via orphan nuclear receptor SHP-mediated transactivation of Runx2. *Bone*.

[37] Gao Y., Li Y., Xue J., Jia Y., Hu J. (2010). Effect of the anti-diabetic drug metformin on bone mass in ovariectomized rats. *European Journal of Pharmacology*.

[38] Min K. S., Lee Y. M., Hong S. O., Kim E. C. (2010). Simvastatin promotes odontoblastic differentiation and expression of angiogenic factors via heme oxygenase-1 in primary cultured human dental pulp cells. *Journal of Endodontics*.

[39] Kim J. K., Shukla R., Casagrande L. (2010). Differentiating dental pulp cells via RGD-dendrimer conjugates. *Journal of Dental Research*.

[40] Kim I. S., Song Y. M., Hwang S. J. (2010). Osteogenic responses of human mesenchymal stromal cells to static stretch. *Journal of Dental Research*.

[41] Lu Y., Ye L., Yu S. (2007). Rescue of odontogenesis in *Dmp1*-deficient mice by targeted re-expression of DMP1 reveals roles for DMP1 in early odontogenesis and dentin apposition in vivo. *Developmental Biology*.

[42] Posa F., di Benedetto A., Colaianni G. (2016). Vitamin D effects on osteoblastic differentiation of mesenchymal stem cells from dental tissues. *Stem Cells International*.

[43] Jin B., Choung P. H. (2016). Recombinant human plasminogen activator inhibitor-1 accelerates odontoblastic differentiation of human stem cells from apical papilla. *Tissue Engineering Part A*.

[44] Zou H., Wang G., Song F., Shi X. (2017). Investigation of human dental pulp cells on a potential injectable poly(lactic-co-glycolic acid) microsphere scaffold. *Journal of Endodontics*.

[45] Egbuniwe O., Idowu B. D., Funes J. M., Grant A. D., Renton T., Di Silvio L. (2011). P16/p53 expression and telomerase activity in immortalized human dental pulp cells. *Cell Cycle*.

[46] Goto N., Fujimoto K., Fujii S. (2016). Role of MSX1 in osteogenic differentiation of human dental pulp stem cells. *Stem Cells International*.

[47] Linde A., Goldberg M. (1993). Dentinogenesis. *Critical Reviews in Oral Biology and Medicine*.

[48] Han Q. Q., Du Y., Yang P. S. (2013). The role of small molecules in bone regeneration. *Future Medicinal Chemistry*.

[49] Segar C. E., Ogle M. E., Botchwey E. A. (2013). Regulation of angiogenesis and bone regeneration with natural and synthetic small molecules. *Current Pharmaceutical Design*.

